# MIF, secreted by human hepatic sinusoidal endothelial cells, promotes chemotaxis and outgrowth of colorectal cancer in liver prometastasis

**DOI:** 10.18632/oncotarget.4198

**Published:** 2015-06-02

**Authors:** Chun-Ting Hu, Li-Li Guo, Na Feng, Lei Zhang, Na Zhou, Li-Li Ma, Lan Shen, Gui-Hui Tong, Qian-Wen Yan, Shi-Jie Zhu, Xiu-Wu Bian, Mao-De Lai, Yong-Jian Deng, Yan-Qing Ding

**Affiliations:** ^1^ Department of Pathology, Nanfang Hospital and School of Basic Medical Sciences, Southern Medical University, Guangzhou 510515, China; ^2^ Guangdong Provincial Key Laboratory of Molecular Tumor Pathology, Guangzhou 510515, China; ^3^ Department of General Surgery, Renji Hospital, Shanghai Jiaotong University School of Medicine, Shanghai 200127, China; ^4^ Department of Pathology, Southwest Hospital, Third Military Medical University, Chongqing 400038, China; ^5^ Department of Pathology, School of Medical Sciences, Zhejiang University, Hangzhou 310006, China

**Keywords:** colorectal cancer, hepatic sinusoidal endothelial cell, macrophage migration inhibitory factor, chemotaxis, metastasis

## Abstract

Growth and invasion of metastatic colorectal cancer (CRC) cells in the liver depend on microenvironment. Here, we showed that human hepatic sinusoidal endothelial cells (HHSECs) induce chemotaxis and outgrowth of CRC cells. Macrophage migration inhibitory factor (MIF), released by HHSECs, stimulated chemotaxis of CRC cells. MIF secreted by HHSECs, but not by CRC cells themselves, promoted migration and epithelial-mesenchymal transition (EMT) and facilitated proliferation and apoptotic resistance of CRC cells. In orthotopic implantation models in nude mice, exogenous MIF stimulated growth of CRC cells and metastasis. Furthermore, MIF accelerated mobility of CRC cells by suppressing F-actin depolymerization and phosphorylating cofilin. Noteworthy, MIF levels were correlated with the size of hepatic metastases. We suggest that HHSECs and paracrine MIF promote initial migration and proliferation of CRC cells in the hepatic sinusoids to generate liver metastases.

## INTRODUCTION

Hepatic metastasis is the leading cause of death in patients with colorectal cancer (CRC), and approximately one-third of CRC patients will develop liver metastases within 3 years after diagnosis [1]. Only 25% of patients have isolated hepatic metastases that can be resected curatively, and 21–48% survive more than 5 years with low mortality [2–4]. The main cause of treatment failure and death is the formation of metastases [5]. Circulatory dissemination of CRC cells to the liver occurs via the portal vein system [6]. Cancer cells get arrested in capillaries of a similar diameter to that of the cells, and extravasation typically occurs in small capillaries [7–9]. They can roll on the endothelium under flow conditions *in vitro*. Yet the rolling has not yet been described *in vivo* in capillaries in the liver [9–12].

Circulating cancer cells usually extravasate and then start to proliferate in the stroma. However, in some cases (for example, in the liver) they initially proliferate in the blood vessels, then cross the endothelium and invade the underlying tissues as groups [7, 9]. So, in the hepatic microvasculature, CRC cells are in a prometastatic condition. It is possible that endothelial cells recruit prometastatic cancer cells, supporting their survival and proliferation. Prometastatic cancer cells that survive in the liver microvasculature can communicate with the cells in the liver, such as human hepatic sinusoidal endothelial cells (HHSECs), Kupffer cells, inflammatory cells, stellate cells and hepatocytes, etc. Soluble paracrine and juxtacrine factors released or induced by these cells play a role in liver metastasis [13–20].

The microenvironment is capable of normalizing cancer cells [21], suggesting that targeting stromal cells, rather than cancer cells themselves, may be an alternative strategy for cancer treatment [19, 20, 22, 23]. Here we explore the seed and soil model and interaction between CRC cells and intrahepatic cells, including the stroma and parenchyma cells. We found that HHSECs mediate CRC cell migration. A protein array assay detected macrophage migration inhibitory factor (MIF), which was secreted in culture medium of HHSECs, particularly when they were adjacent to CRC cells. The purpose of this study was to understand the role of HHSECs and their secreted MIF in mediating the chemotaxis of prometastatic CRC cells.

## RESULTS

### HHSECs induce chemotaxis during CRC cell migration

We first assessed whether normal cells originating from the liver and non-specific target organs exerted differential effects on the migration of CRC cells. A Transwell assay was utilized to compare the attractant ability toward CRC cell migration, wherein human normal cells were placed in the bottom chamber, and CRC cells (SW480, HCT116, or LS174T) were placed in the upper chamber. The normal cells of the liver included HHSECs, HL7702s (human hepatocytes), and LX-2s (human hepatic stellate cells), and corresponding cells including HUVECs (human umbilical vein endothelial cells), 293As (human embryonic kidney cells), and BJs (human foreskin fibroblast cells) were compared as analog-control cells originating from non-specific target organs of CRC metastasis. This model simulates the prometastatic cancer cells in the liver sinusoids chemotracted by the adjacent cells.

**Figure 1:**
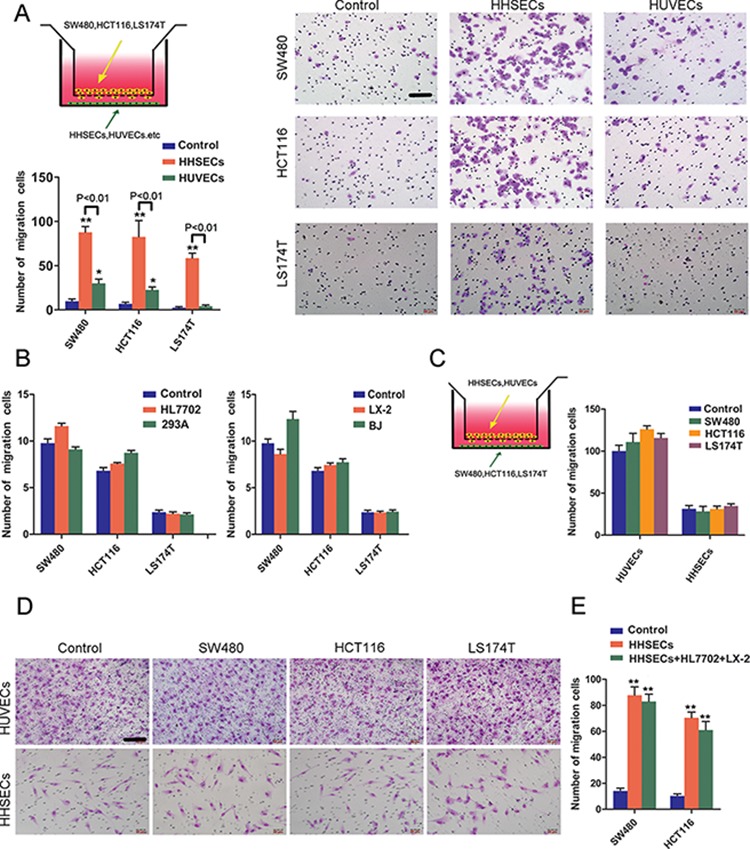
HHSECs induced CRC cell chemotaxis in the Transwell model **A.** Transwell co-culture model and chemotaxis of each CRC cell type toward HUVECs or HHSECs (compared to controls), and representative images of migrated CRC cells chemotracted by HHSECs or HUVECs. The co-cultured cells on the top and bottom of the Transwell chamber were not in direct contact. Scale bar, 100 μm. **B.** Transwell migration activity of CRC cells induced by HL7702 or 293A, and LX-2 or BJ (compared to controls). **C.** The CRC cell position was reversed in the Transwell chamber to chemotract HUVECs or HHSECs; results are shown compared to the respective control. **D.** Representative images of migrated HUVECs or HHSECs attracted by CRC cells. Scale bar, 100 μm. **E.** HHSECs, and HL7702 and LX-2 cells mixed together, or HHSECs alone induce CRC cells migration. Data are means ± *SD* from three independent experiments. *P < 0.01 or **P < 0.001 compared with controls. *P* < 0.01 between groups.

The results showed that HHSECs were 3 to 14 times more active than HUVECs in stimulation of CRC cells migration (Figure 1A). HL7702, 293A, LX-2, and BJ cells induced the migration of CRC cells in a way that was not obviously different from that of the controls (Figure 1B), and the cells that originated from the target organ (liver), such as HL7702 and LX-2, did not show any positive differential roles in promoting migration of CRC cells, but had similar effects to those of the non-target organ cells, such as 293A and BJ.

Subsequently, when the cell positions were reversed in the Transwell chamber, the HHSECs, HUVECs, HL7702, and LX-2 in the upper chamber were not chemotracted by CRC cells in the bottom chamber (Figure 1C and 1D, Supplementary Figure S1A). Furthermore, when HHSECs, and HL7702 and LX-2 cells were mixed in a co-cultured system to induce CRC cell migration, the chemoattractant effect of the mixed cells was not much greater than that of HHSECs alone (Figure 1E). In addition, we also tried to demonstrate whether another tumor cell that metastasizes to the liver as a specific target organ, HCC1937s (human breast cancer cells), used as a positive control, was attracted by HHSECs or HL7702 or LX-2 cells. We used RL95s (endometrial cancer cells) as the negative control, as it rarely metastasizes to the liver. Interestingly, HHSECs induced HCC1937 migration more markedly than that of RL95 (Supplementary Figure S1B), but neither the breast nor endometrial cancer cell lines chemotracted HHSECs or HUVECs to migrate (Supplementary Figure S1C). Thus, the Transwell assays demonstrated that HHSECs were the dominant cells for chemotracting CRC cells to metastasize to the liver.

### MIF is a critical factor released by HHSECs and contributes to the chemotaxis of CRC cell migration

**Figure 2:**
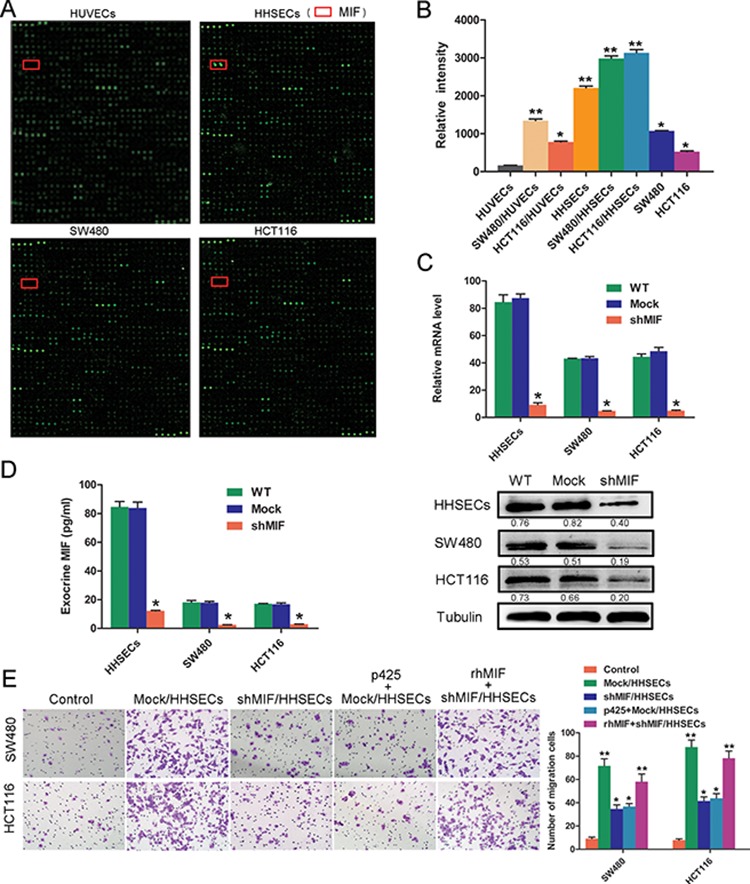
MIF secreted by HHSECs is a critical factor for CRC cell migration **A.** Protein array assay (Raybiotech) using conditioned media of HUVECs or HHSECs, or SW480 or HCT116 cells. The positions of MIF are indicated by red frames. **B.** Protein array data of MIF in different conditioned media. *P < 0.01, **P < 0.001 compared with HUVEC conditioned media. **C.** MIF mRNA expression analyzed by RT-qPCR and western blotting following MIF knockdown. *P < 0.01, compared with WT. WT = wild type, Mock = lentiviral vectors without shMIF. **D.** MIF secreted by cells as detected by ELISA. *P < 0.01 compared with WT. **E.** Transwell migration activity of SW480 and HCT116 cells induced by different conditioned media and their representative images. *P < 0.01, **P < 0.001 compared with the control. Data are means ± *SD* from three independent experiments.

To ascertain which mediator(s) might be released from HHSECs to induce CRC cell migration, we compared the culture supernatants (conditioned media) that were collected from the upper and lower chambers of the Transwell dish by using human cytokine arrays containing antibodies against 1000 cytokines. Analysis of the antibody array demonstrated that MIF, IGFBP-7, Smad 4, SPARC, thrombospondin (TSP), and Ras are mediators whose expression levels are significantly higher in HHSECs than in HUVECs and CRC cells (Supplementary Figure S2A). Among these proteins, MIF showed the greatest expression in the conditioned media from HHSECs, particularly in SW480/HHSECs and HCT116/HHSECs, in comparison with that from HUVECs, and SW480 and HCT116 cells (Figure 2A and 2B, Supplementary Figure S2B). Application of either of two specific MIF inhibitors (ISO-1 and P425) resulted in the inhibition of HHSEC-induced migration of CRC cells. As a positive control, rhMIF (human recombinant MIF) was confirmed to promote CRC cell migration. To assess the chemotactic significance of MIF from HHSECs, MIF expression was inhibited by lentiviral vector-mediated small hairpin (shRNA) expression in HHSECs. Quantitative real-time PCR (qPCR), western blot (WB) (Figure 2C), and enzyme-linked immune sorbent assay (ELISA) (Figure 2D) verified the efficacies of MIF knockdown and MIF secretion blockage.

We found that Mock/HHSECs treated by the MIF inhibitor p425 (100 nM) or shMIF/HHSECs resulted in the inhibition of HHSEC-induced migration, but that the inhibitory effect could be recovered by supplementation with rhMIF (50 nM) (Figure 2E). To explore whether MIF was released by HHSECs or whether MIF in the CRC cells themselves could act as a main factor in migration, we knocked down MIF in SW480 and HCT116 cells. Mock/HHSECs chemotracted shMIF/SW480 or shMIF/HCT116 to migrate was markedly increased, in comparison with shMIF/HHSECs or Mock/HHSECs plus p425 chemotracted (Supplementary Figure S2C and S2D). We also utilized WB and ELISA to detect whether the MIF was expressed or secreted by other metastatic microenvironmental cells including HL7702 and LX-2 cells and HUVECs. HL7702s, LX-2s, and HUVECs also expressed intracellular MIF, and hardly excreted MIF (Supplementary Figure S2E and S2F). The mRNA coding sequence of MIF in HHSECs was the same as that in SW480, HCT116, and HUVECs as assessed by reverse transcription (RT)-PCR amplification (Supplementary Figure S2G). Thus, these results suggested that the MIF released from HHSECs is a major mediator contributing to the HHSEC-induced migration of CRC cells.

### MIF released by HHSECs promotes the epithelial-mesenchymal transition (EMT), proliferation, and apoptotic resistance of CRC cells

**Figure 3:**
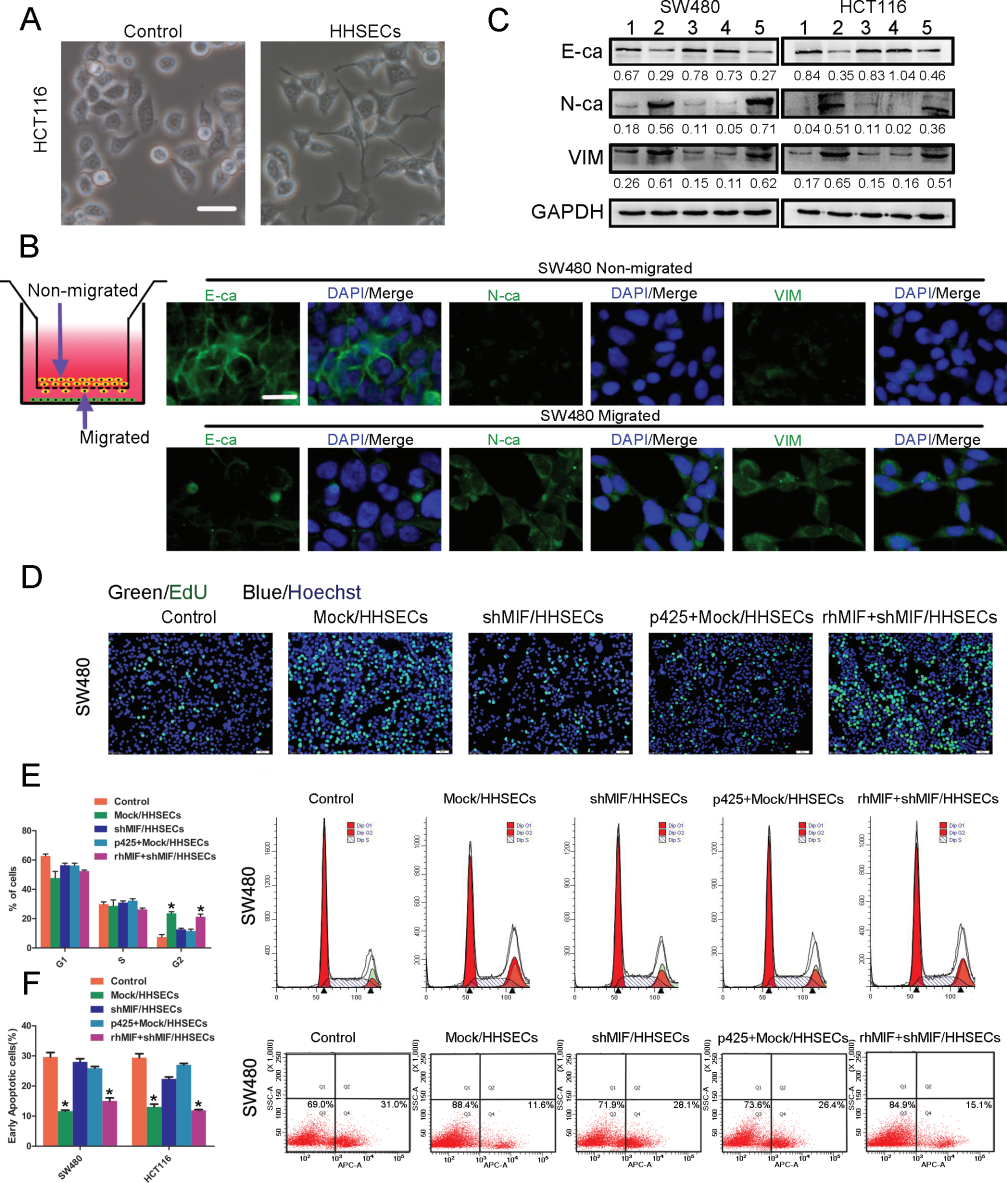
MIF released by HHSECs promotes the EMT, proliferation, and apoptotic resistance of CRC cells **A.** Bright-phase microscopy: Cell protuberances of HCT116s induced by conditioned media collected from HHSEC culture. Scale bar, 20 μm. **B.** Transwell chemotactic model of migrated and non-migrated cells. Immunofluorescence: migrated and non-migrated cells of SW480 chemotactic by HHSECs expression of E- and N-cadherin and vimentin. Scale bar, 20 μm. **C.** Immunoblot: effect of MIF on activation of CRC cells expressing E- and N-cadherin and vimentin after being cultured with conditioned media. 1. Basic medium, 2. Conditioned medium from Mock/HHSECs culture, 3. Conditioned medium from shMIF/HHSECs culture, 4. p425 added in conditioned medium from Mock/HHSECs culture, 5. rhMIF added in conditioned medium from shMIF/HHSECs culture. **D.** EdU (5-ethynyl-2′-deoxyuridine) cell proliferation assay of SW480. **E.** Cell cycle analysis of SW480 grown in different conditioned media. MIF induced G2 phase arrest in SW480. *P < 0.01 compared with control. **F.** 5-FU-induced apoptosis was inhibited in SW480, which was cultured with conditioned media containing MIF. *P < 0.01 compared with control. Data are means ± *SD* from three independent experiments.

When CRC cells were cultured with conditioned media from HHSECs, the CRC cells appeared starfish shaped, which were composed of cytoplasmic protuberances (Figure 3A). The Transwell migration model was used to determine whether the EMT that occurred within the CRC cells exhibited chemotaxis towards HHSECs. We found that the migrated CRC cells induced by HHSECs strongly expressed mesenchymal products such as N-cadherin (N-ca) and vimentin (VIM), and underexpressed epithelial products such as E-cadherin (E-ca) in comparison with non-migrated CRC cells (Figure 3B and Supplementary Figure S3A). To confirm whether EMT was stimulated by MIF released from HHSECs, the CRC cells were cultured with different conditioned media for 24 hours. WB analysis showed that the CRC cells exhibited elevated N-ca and VIM expression but lost E-ca when grown in conditioned media containing higher levels of soluble MIF (Figure 3C).

The proliferative effect of paracrined MIF on CRC cells was assessed with a CCK8 assay, following treatment of SW480 and HCT116 cells with different conditioned media. The CCK8 assay results indicated that MIF released at high concentrations from HHSECs was favorable to CRC cell proliferation (Supplementary Figure S4A), which was similar to the result of the EdU (5-ethynyl-2′-deoxyuridine) assay (Figure 3D and Supplementary Figure S3B).

To explore the mechanism by which CRC cell proliferation was promoted by exogenous MIF, we studied the effect of MIF on the cell-cycle phases using flow cytometer. The percentage of cells in G2 phase was significantly increased by the presence of MIF in the conditioned media, while no significant effect on the percentage of cells in G1 or S phase was observed (Figure 3E and Supplementary Figure S3C). Furthermore, Annexin V staining as an indicator of apoptosis was used to measure the apoptotic rates in association with the proliferative effect. This analysis demonstrated that soluble MIF inhibited the apoptosis of CRC cells induced by 5-fluorouracil (5-FU) (Figure 3F and Supplementary Figure S3D). Collectively, these data implied that MIF released by HHSECs activated EMT, proliferation, and apoptotic resistance during CRC cell migration.

### MIF released by HHSECs facilitates CRC growth and migration *in vivo*

**Figure 4:**
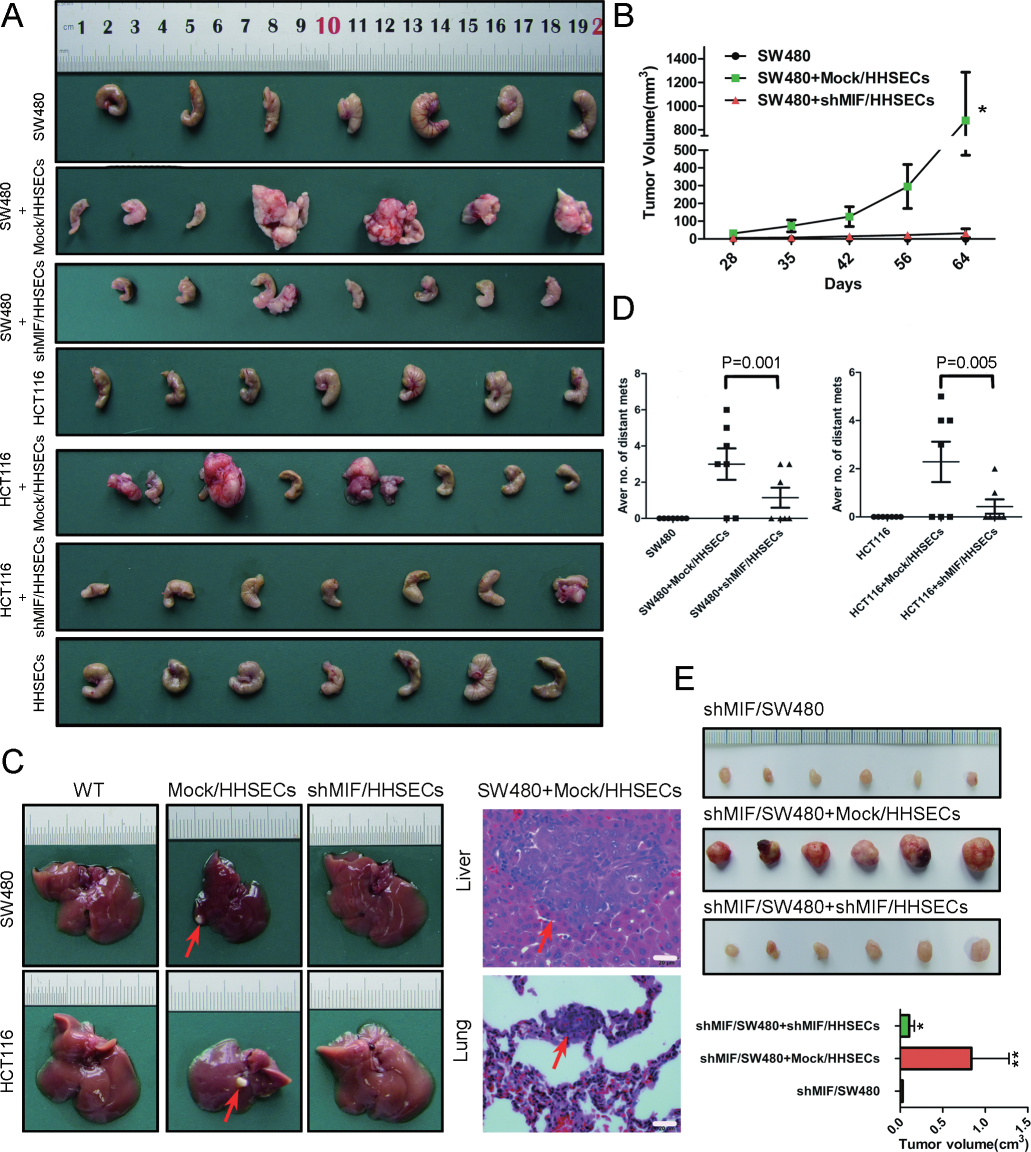
MIF increases the stimulatory effects of HHSECs on CRC cell umorigenesis and metastasis **A.** Cecal tumors of nude mice injected with CRC cells or a mixture of CRC cells and different HHSECs. **B.** Growth curve of orthotopic implantation tumors. *P < 0.001 versus cecal injection with SW480 alone. **C.** Liver metastatic nodules (red arrow) after injection of the CRC cells in combination with Mock/HHSECs into the cecal wall and representative H&E-stained sections of the liver and lung tissues. Red arrows point to the tumor foci. **D.** Quantitative analysis by counting the tumor foci in the livers and lungs under microscopic examination. **E.** Photographs of subcutaneous tumors in the mice injected with shMIF/SW480 alone or in combination with Mock/HHSECs or shMIF/HHSECs and the analysis of subcutaneous tumors. A robust increase can be observed in tumor volume due to Mock/HHSECs co-implantation, which is abolished with MIF knockdown. *P < 0.05, **P < 0.001 versus shMIF/SW480 alone.

Orthotopic transplantation of nude mice to generate experimental metastasis was utilized to ascertain whether the MIF released from HHSECs increases the growth, invasiveness, and liver metastases of CRC cell-derived tumors. CRC cells, or CRC cells mixed with Mock/HHSECs, CRC cells mixed with shMIF/HHSECs, and HHSECs alone, were implanted into the cecal wall of nude mice for 8 weeks. The mice were euthanized and subjected to gross and microscopic examination. Gross and microscopic examination of hepatic and pulmonary metastases revealed that the tumor growth of the CRC and Mock/HHSEC cells mixture at the primary site was dramatically increased (Figure 4A) and that the tumor growth rate, volume, weight, and foci were markedly higher than those of the tumors of CRC cells alone or of CRC cells mixed with shMIF/HHSECs (Figure 4B–4D). However, HHSECs injected alone did not generate any masses.

The nude mice cecum hardly developed primary tumors by implanted CRC cells alone. Therefore, we compared the proliferation and apoptosis in the other two groups. Immunohistochemistry for the proliferation marker Ki-67 revealed a higher degree of proliferation in primary tumors arising from implantation of SW480 mixed with Mock/HHSECs than from SW480 mixed with shMIF/HHSECs (*P* = 0.001); no significant difference was observed in liver metastasized tumors (Supplementary Figure S4B and S4C). In contrast, the expression of caspase3, which is a key cellular protein that triggers the apoptosis process, was found to be opposite to that of Ki-67, although there also was no significant difference in liver metastatic tumors for this protein (Supplementary Figure S4C).

Additionally, shMIF/SW480 cells, shMIF/SW480 cells mixed with Mock/HHSECs, or shMIF/SW480 cells mixed with shMIF/HHSECs were also subcutaneously implanted into nude mice. This revealed that Mock/HHSEC cells with MIF secretion activated tumorigenesis and tumor growth (Figure 4E). In summary, these results suggest that MIF secreted from HHSECs promotes tumorigenesis and the development of CRC cell metastases *in vivo*.

### MIF paracrined from HHSECs induces CRC cell migration through p-cofilin to increase F-actin polymerization

**Figure 5:**
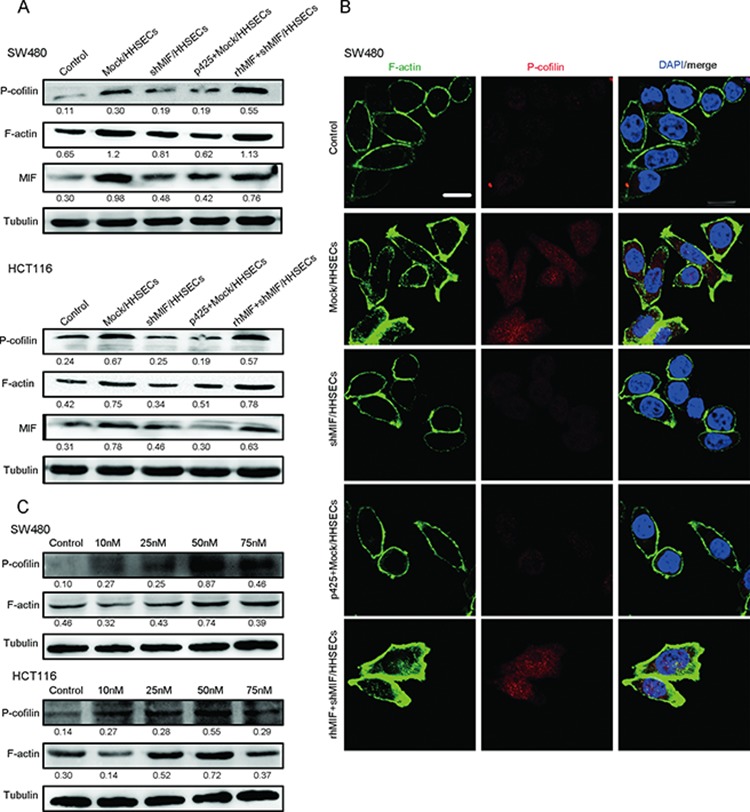
MIF released by HHSECs regulates cytoskeletal proteins **A.** Western blotting analysis of p-cofilin, F-actin, and MIF expression in CRC cells cultured with different conditioned media. **B.** Immunofluorescence images of p-cofilin and F-actin expression in CRC cells. Scale bar, 10 μm. **C.** Western blot analysis of p-cofilin, F-actin, and MIF expression in CRC cells stimulated with rhMIF at the indicated concentrations.

To determine whether the signaling pathways involved in paracrine MIF also induce the migration of CRC cells, cells were cultured with conditioned media as previously described. We identified that p-cofilin expression in the CRC cells was more marked when cells were cultured in the conditioned media from Mock/HHSECs than in other media (conditioned media from shMIF/HHSECs, Mock/HHSECs supplementing p425, and fundamental medium). However, p-cofilin expression was restored following cultivation in the conditioned media of shMIF/HHSECs supplemented with rhMIF (Figure 5A and Supplementary Figure S5A). Phosphorylation inactivates cofilin, leading to the accumulation of actin filaments [24]. As shown by WB and immunofluorescence, intracellular F-actin was more prominent when the cells were cultured by the conditioned media of Mock/HHSECs, but the enhanced expression was offset following cultured in the conditioned media of shMIF/HHSECs or Mock/HHSECs plus p425 (Figure 5B). To confirm whether signaling of CRC cells by secreted MIF leads to cofilin phosphorylation and is involved in F-action regulation, we treated CRC cells with rhMIF at the indicated concentrations. As shown in Figure 5C, addition of rhMIF at 50 nM led to increased p-cofilin and F-actin expression, whereas addition of rhMIF at concentrations greater than 50 nM did not promote further expression. The characteristic effects of MIF derived from HHSECs on the cofilin/F-actin cytoskeleton suggested that it might be specifically involved in the cytoskeletal remodeling that promotes CRC migration.

### MIF is associated with the metastatic outgrowth of CRC

**Figure 6:**
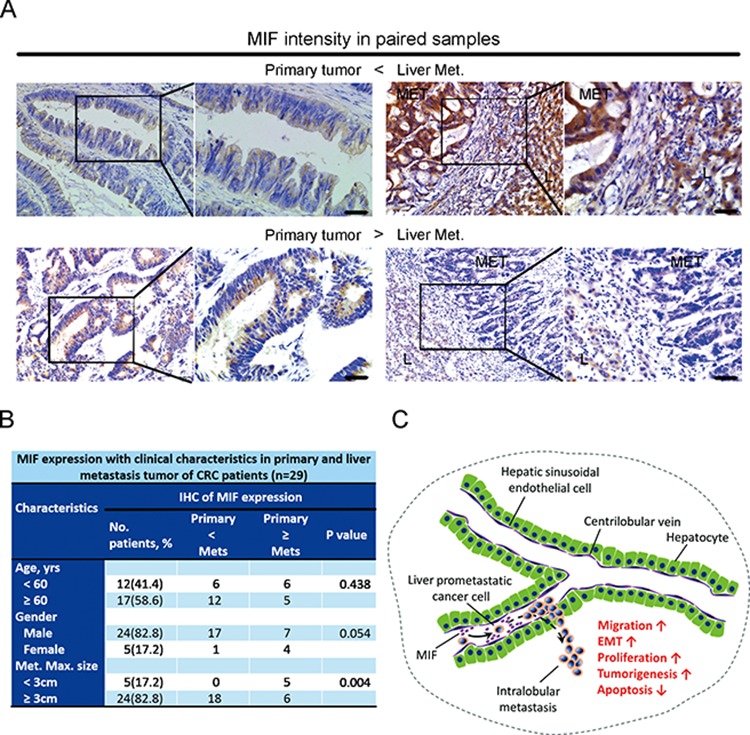
Analysis of tumors in patients with CRC and a proposed model of secreted MIF-mediated chemotraction of CRC cells in the liver sinusoids **A.** Immunohistochemistry: representative images of MIF intensity in paired samples. Scale bar, 40 μm. Met, metastasis; L, liver. **B.** MIF expression with associated clinical characteristics. **C.** Proposed model for MIF-mediated paracrine activation of CRC cell migration and other functions during liver prometastasis.

We used immunohistochemistry staining to examine the MIF protein expression in primary CRC samples from 229 patients collected from the Pathology Department of Nanfang Hospital, Southern Medical University, Guangzhou, China. There was no statistically significant correlation between MIF expression levels and cancer invasion, histological grades, survival time, or lymph node or distant metastases (Supplementary Table S1). Therefore, the MIF expression levels that originated from CRC cells or from other undefined cells in the primary tumor microenvironment did not appear to have an important prometastatic effect. We also used immunohistochemistry to examine the MIF expression in paired samples of tubular adenocarcinoma from 29 patients with CRC in primary tumors and liver metastases. According to the distinct expression of MIF in primary tumors from metastatic tumors, we classified all samples into two groups. Group A included the samples that expressed MIF in the primary cancer tissues at levels less than in the liver metastases, while Group B included the samples that expressed MIF in primary cancer tissues at levels greater than or equal to that of the liver metastases (Figure 6A). Of the 29 paired samples that were analyzed, 0 (0%) patients in Group A and 5 (45%) in Group B produced liver metastases with a maximum size less than 3 cm. Approximately 18 (100%) patients in Group A and 6 (55%) in Group B produced liver metastases with a maximum size greater than or equal to 3 cm (*P* < 0.05). There were no statistically significant differences in age or gender between Groups A and B (*P* > 0.05, Figure 6B). These results suggested that the sizes of the liver metastases were highly positively correlated with the expression of MIF, and that HHSECs had a promoting effect.

## DISCUSSION

Organ-specific metastasis of the liver is the main cause of treatment failure and death for CRC patients [5]. Two major theories organ-specific metastasis: the target organ specifically presenting chemoattractants that allure tumor cells to settle down [5, 25], and cancer cells interact with the microenvironmental factors of the host for growth advantage [5, 26]. Endothelial cells also promote the formation of a three-dimensional profile between cancer and stromal cells [19, 27]. Besides their role in increasing blood flow and nutrient delivery to tumors, endothelial cells also express factors (termed angiocrine factors) that might promote tumor progression and therapeutic resistance [28].

Bone marrow-derived endothelial and hematopoietic precursor cells enhance metastasis of cancer [29, 30]. Circulating cancer cells enter the hepatic microvasculature and may initially proliferate in the blood vessels, then cross the endothelium and invade the underlying tissues [7, 9]. However, once the circulating CRC cells enter the liver, the CRC cells should be adjacent to HHSECs, then extravasate to hepatic stellate cells and hepatocytes in accordance with the hepatic architecture [25, 31, 32].

Here we showed that CRC cells did not contact with HHSECs. We observed that HHSECs, but not hepatic stellate cells (LX-2) and hepatocytes (HL7702), were the dominant cells that induced chemotaxis of CRC cells. Non-target organ cells, including HUVECs, human embryonic kidney cells (293A) and human foreskin fibroblast cells (BJ), did not induce chemotaxis with CRC cells. We also found that prometastatic CRC cells revolved around the HHSECs because CRC cells did not chemotracted HHSECs migration. These indicated that target organ-derived endothelial cells in the liver were the dominant cells for CRC cell migration.

The conditioned medium of HHSECs induced protrusive structures of CRC cells. The mechanism of CRC cell migration attracted by HHSECs should be associated with some molecules and possible pathways. This indicated that HHSECs might secrete some factors to stimulate the CRC cells. Human cytokine arrays revealed that MIF produced by HHSECs was a critical molecule that participated in CRC cell migration, and while MIF was knocked down or specifically blocked in HHSECs, the chemoattractant capacity of HHSECs to CRC cells was dramatically depressed. Another example has shown that the pathway of planar cell polarity is activated in breast cancer cells by a soluble factor derived from fibroblasts which conditioned media from the mouse L fibroblast cell line induced protrusive structures and motility of human breast adenocarcinoma MDA-MB-231 cells [33]. Therefore, paracrine MIF from HHSECs is critical to induce protrusive structures and chemotaxis of the intra-sinusoidal prometastatic CRC cells.

MIF was originally known as a secreted proinflammatory cytokine with an effect on innate immunity, and it has a broad distribution in normal tissue, including the gastrointestinal tract and liver (Kuppfer cells, hepatocytes and endothelial cells) [34]. MIF might contribute to the genomic instability within tumors, as MIF suppresses p53 function [35], potentially leading to the attenuation of normal apoptosis and growth-arrest [36]. MIF is also associated with multiple disorders, including autoimmunity, obesity and cancer [37]. It has the functional role of tumor promoter in the inflammation-tumorigenesis axis [26, 38, 39]. The cytoplasmic expression of MIF was found in the tumor cells of the lung, breast, liver, colon, and prostate [1, 40–42]. MIF plays the role of controlling the processes of cell proliferation, differentiation, apoptosis, invasion, angiogenesis and metastasis [43].

Previous reports of tumor-derived MIF enhanced the progression of renal cell carcinoma and intestinal tumorigenesis [44, 45]. Our data suggested a role for MIF paracrine from HHSECs in the prometastatic stage of CRC, as well as other biological functions. The significantly facilitated CRC growth, increased the cell percentage of G2 phases, and promoted apoptotic resistance and EMT *in vitro*. A newly technique showed that growth rates increase with progression through the cell cycle and reach a maximum during G2 in very different cell lines [46], which is consistent with a recent study using a newly developed optical interferometric technique to measure cycle-dependent of cell growth [47]. In addition, the proliferative assay of CCK8 and EdU showed that paracrine MIF promotes CRC cells proliferation, therefore, we thought that paracrine MIF increased the CRC cells growth rates through controlling the cells transit to G2 phase.

Most importantly, in the experimental metastasis of nude mice orthotopic transplantation, we set up the control groups to exclude the MIF secreted from other cells, even though MIF released from the microenviroment at a base level, the prometastatic effect of adding Mock/HHSECs in the co-transplanted cells was distinctly enhanced in a relatively short time. To exclude the effect of MIF from CRC cells themselves, we also knockdown the expression of MIF in CRC cells, the MIF secreted by HHSECs promoted tumorigenesis and tumor growth markedly in nude mice co-transplanted Mock/HHSECs subcutaneously. Therefore, MIF secreted from HHSECs play a pivotal role of promoting outgrowth and prometastatic effect compared with other MIF-secreting cells including CRC cells themselves.

We also used IHC to examine the MIF expression levels in primary CRC samples of 229 patients. There were no statistical significant correlation of MIF expression levels with the prognosis and distant metastasis. So MIF derived from CRC cells, or some other undefined cells in primary tumor microenvironment, did not play a prometastatic effect. Interestingly, MIF expression was positively related to the tumor size of live metastases. These suggest liver metastases were positively correlated with the expression of MIF, and HHSECs had a promoting effect.

MIF might mediate its biological activities either through a classical receptor-mediated pathway, which is mediated by CD74 and CD44 receptors [48, 49], or through a non-classical endogenous pathway. In CRC cells cultured with conditioned media that contained or did not contain exogenous MIF, we observed that the expression of CD74 and CD44 had conspicuously changed (Supplementary Figure S5B). Therefore, our data was in accordance with a model in which CD74 forms a signaling complex with CD44 to mediate the MIF functions. In fact, MIF secreted by HHSECs acted as a positive regulator with the cofilin/F-actin pathway by altering the expression of p-cofilin in CRC cells. Cell motility depends on the regulated dynamics of the actin cytoskeleton [50, 51], we also found that the increased polymerization of F-actin in the CRC cells that was related to cell migration. Therefore, the crosstalk between the endothelial cells of the target organ and the seeding cells was mediated by a soluble factor of MIF.

Here, we found that HHSECs and their secreted MIF were the dominant cells and the key molecule for CRC prometastatic chemotaxis in the liver (Figure 6C). We noted that HHSECs and their paracrine MIF might be a target for anti-soil and anti-seed therapy, and provided antagonists of concept for migration and proliferation of CRC cells during prometastasis.

## MATERIALS AND METHODS

### Cell culture

Human CRC cell lines (SW480, HCT116, LS174T), HCC1937 and human HL7702 were cultured in RPMI-1640 (Gibco). LX-2 and 293A were cultured in DMEM with high glucose (Gibco). HUVECs and RL95 were maintained in DMEM/F-12 (Gibco). BJ was cultured in Minimum Essential Medium (Gibco). HHSECs were cultured in Endothelial Cell Medium plus ECGS (ScienCell) and 100 units/mL of Penicillin/Streptomycin Solution (ScienCell). All cells were supplemented with 5% or 10% fetal bovine serum (Gibco) and incubated in a humidified atmosphere with 5% CO_2_ at 37°C. HCC1937 and RL95 were obtained from the cell bank of the Chinese Academy of Sciences (CAS). HHSECs were purchased from ScienCell Research Laboratories in USA. All other cells were maintained by the Guangdong Provincial Key Laboratory of Molecular Tumor Pathology, Guangzhou, China.

### Cell migration assays

The migration assays were performed in 24-well cultured plates with 8 μm pore size transwell chamber inserts (BD Biosciences). Chemotactic cells (1 × 10^5^ cells/well) were added to the bottom chambers of 24-well culture plates, when the cell incubation with adherence, the cultured medium replaced by RPMI-1640 (0.75 mL) containing 0.2% fetal bovine serum (FBS), and the cells (2 × 10^5^/mL) were induced to migrate, filled with RPMI-1640 (0.25 mL) containing 0.2% FBS, and added to the upper chamber, while the cells in the upper and bottom chamber were not in contact. Cell migration in the control group was determined by placing RPMI-1640 (0.75 mL) containing 0.2% FBS in the bottom chamber. After incubation at 37°C / 5% CO_2_ for 24 hours, the non-migrated cells that remained on the upper surface of the membrane were scraped. The migrated cells on the lower face of the membrane were fixed with chilled methanol and stained with Giemsa stain solution. Migrating cells were counted under a light microscope from 10 random fields at 200× magnification for each triplicate sample.

### Immunofluorescence of epithelial mesenchymal transition

In migration assays, the non-migrated or migrated cells on the membranes of the transwell chamber inserts were scraped in order to obtain the migrated or non-migrated cells, and the cells on the membrane were fixed with 4% paraformaldehyde and permeabilized with 0.3% Triton X-100 prior to blocking with 10% goat serum. The primary antibodies of E-cadherin (1:100, EP700Y, Epitomics), N-cadherin (1:100, EPR1791–4, Epitomics), and Vimentin (1:100, EPR3776, Epitomics) were incubated overnight at 4°C. Secondary antibodies were goat anti-mouse or -rabbit IgG coupled to Alexa-488 or -594. Cell nuclei were stained with DAPI (Sigma).

### Protein array assay

The culture media in the upper (0.25 mL) and lower (0.75 mL) transwell chambers were harvested after the cells migrated for 24 hours. Approximately 1 mL of cell medium was collected from SW480, SW480/HHSECs, SW480/HUVECs, HCT116, HCT116/HHSECs, HCT116/HUVECs, HHSECs, and HUVECs. The collected supernatants were centrifuged at 1000 g for 10 min and the protein expression profiles were analyzed using Human Antibody Array 1000 (Raybiotech, AAH-BLG-1000, USA), which detects 1000 cytokines. The protocol was performed in accordance with the instructions of the manufacturer. Signal intensity was measured by spot densitometry using streptavidin-conjugated HiLyte Fluor 532™ for fluorescence detection. The complete array maps can be found at http://www.raybiotech.com/human-l-1000-array-glass-slide-2.html.

### Knockdown of MIF by lentiviral vector

To establish a stable knockdown of MIF, the HHSECs, SW480 and HCT116 were infected with lentivirus expressing shRNA against MIF. The shRNA was designed and packaged by GenePharma (Shanghai, China). The sequences of the two cDNA fragments (sense strands) are as follows: MIF, 5′-TGCACAGCATCGGCAAGAT-3′. The transfection of the cells with the virus was performed according to the manufacturer's instructions.

### MIF detection of ELISA

Human MIF Quantikine ELISA Kits (R&D Systems) were used according to the directions of the manufacturer. Approximately 200 μL of conditioned media, which cultured the cells from HHSECs, Mock/HHSECs, shMIF/HHSECs, SW480, Mock/SW480, shMIF/SW480, HCT116, Mock/HCT116, shMIF/HCT116, HL7702, LX-2 and HUVECs, were collected from triplicate samples.

### Quantitative real-time PCR analysis

Total RNA from cells was extracted and reverse transcribed, and the specific primers for human MIF were designed. The primer sequences were as follows: forward 5′-AGCAGCTGGCGCAGGCCAC-3′ and reverse 5′-CTCGCTGGAGCCGCCGAAGG-3′. Gene expression was normalized to GAPDH. PCR was performed using Ex Taq™ DNA Polymerase (Takara Bio) and an ABI PRISM 7500 Sequence Detection System (Applied Biosystems). Each sample was tested in triplicate.

### Conditioned media preparation

Mock/HHSECs and shMIF/HHSECs (1 × 10^5^ cells) were seeded on 24-well culture plates until cells grew with adherence; the endothelial cell media (ECM) was removed, the cells were washed in sterile PBS, and 1 mL RPMI-1640 with 0.2% FBS was added. Conditioned medium of Mock/HHSECs or shMIF/HHSECs was collected after 24 hours. The conditioned medium was centrifuged for 10 min at 1000 g, and plus p425 (BioVision, 100 nM) in the conditioned medium of Mock/HHSECs or rhMIF (PeProtech, 50 nM) in the conditioned medium of shMIF/HHSECs, preparing the conditioned medium of p425+Mock/HHSECs and rhMIF+ shMIF/HHSECs.

### Western immunoblotting

SW480 and HCT116 cells were seeded in 6-well plates until they grew with adherence, the various conditioned media was replaced as described previously and incubated for 24 hours, and cell extracts were prepared in ice-cold lysis buffer containing protease inhibitor. The cell proteins were separated by SDS-PAGE and blotted onto polyvinylidene difluoride (PVEF) membranes (Millipore). Membranes were further incubated sequentially with specific antibodies including anti-E-cadherin (1:1000, Pro780, Cell Signaling Technology), anti-N-cadherin (1:1000, EPR1791–4, Epitomics), anti-Vimentin (1:1000, EPR3776, Epitomics), anti-MIF (1:1000, Santa Cruz Biotechnology), anti-p-Cofilin (1:1000, 77G2, Cell Signaling Technology), and anti-F-actin (5 μg/ml, 4E3.adl, Abcam). After primary antibodies were incubated, the blots were subsequently incubated with appropriate secondary antibodies. Protein bands were visualized with ECL reagent (Thermo Scientific Inc.) and a Bio-Rad image acquisition system (Bio-Rad Laboratories). The protein bands was quantified using densitometric scanning software, and relative protein abundance was determined by normalization with tubulin or GAPDH.

### Cell proliferation and EdU labeling

CRC cells (1000 cells/well) were seeded in 96-well plates and cultured overnight at 5% CO_2_/37°C, and then cells were treated with different conditioned media for the indicated times. Cell proliferation was detected by using a Cell Counting Kit-8 (Dojindo) every 24 hours. To measure DNA synthesis, CRC cells were stimulated by prepared conditioned medium for 24 hours, and EdU labeling was performed.

### Cell cycle analysis

Approximately 3 × 10^5^ CRC cells per well were treated with conditioned media for 24 hours, collected, and fixed with ice-cold 70% methanol for 30 min. After fixation, cells were pelleted by centrifugation, treated with 20 μg/mL RNase A (Sigma), and stained with 50 μg/mL propidium iodide (Sigma). The stained samples were measured on a FACScan flow-cytometer (Becton-Dickinson).

### Apoptosis assay

SW480 and HCT116 cells were seeded in 6-well plates and treated with different conditioned media containing 5-FU (5 μg/mL). As a positive control, 5-FU was added to basic medium to induce apoptosis. After 24 hours, we utilized an Annexin V Apoptosis Detection Kit to detect the cell surface phosphatidylserine exposure, which serves as an early marker of apoptotic cell death. The samples were analyzed by a FACScan flow-cytometer (Becton-Dickinson).

### Animal model assay

Animal experiments were performed under the guidelines set forth by the Ethics Committee of Medical Research, Southern Medical University, China. For the orthotopic model, we used 4 to 5 week-old athymic BALB/c nude mice. A laparotomy of 1 cm on average was performed and the caecum was isolated. Then, we used a fine needle to inject 50 μL volume of CRC cells (2 × 10^6^) alone, CRC cells combined with Mock/HHSECs, or shMIF/HHSECs (10:1) into the cecal wall; at the same time as the injection, the blister provoked by the inoculation was noted to identify that the serosa had been raised as a result of the injection. The needle was removed and the injection site was inspected to confirm that no leakage had occurred, and the cecum was returned to the abdominal cavity. After 4 weeks following injection, when mice developed signs of distress and we could easily palpate the cecum tumor nodules under the abdominal skin, we measured the tumor size three times with a caliper repeatedly every week. Mice were sacrificed and examined at 8 weeks after injection. For the tumorigenicity assays, tumor cells (2 × 10^6^ cells/mouse) or combinations of mock/HHSECs or shMIF/HHSECs (2 × 10^5^ cells/mouse) were injected subcutaneously into nude mice. The mice were sacrificed 4 weeks after injection. Tumor volume was estimated by the following formula: length × width^2^ × 0.5.

### MIF immunohistochemistry

A total of 229 clinical samples were immunostained with an antibody for MIF (1:100; P14174, ImmunoWay Biotechnology). Slides were deparaffinized and rehydrated, and endogenous peroxidase activity was blocked with 3% H_2_O_2_ for 15 min. After high pressure antigen retrieval using 10 mM sodium citrate buffer (pH 6.0), slides were incubated with the primary antibody overnight at 4°C. Subsequently, sections were incubated for 45 min at room temperature with Poly-HRP anti-/Rb/Ra IgG (DAKO) and DAB reaction (DAKO). The slides were evaluated by three independent observers; all investigators were blinded to the clinical data. The staining intensity was scored as 0 (negative), 1 (weak), 2 (medium), or 3 (strong). The extent of staining was scored as 0 (0%), 1 (1–25%), 2 (26–50%), 3 (51–75%), or 4 (76–100%), according to the percentages of the positive staining areas in relation to the entire carcinoma-involved area. The product of the intensity and the extent scores was used as the final staining score for MIF. Tumors with a final staining score of 8 or higher were considered to have high expression according to distant metastasis classifications with a receiver operating characteristic (ROC) curve.

### Confocal laser scanning microscopy

CRC cells were stimulated with different conditioned media for 24 hours and fixed in 4% paraformaldehyde for 20 min. The cells were incubated with a primary antibody against human p-cofilin (1:100, P23528, ABclonal) or F-actin (5 μg/mL, 4E3.adl, Abcam) overnight at 4°C. After being incubated with the secondary antibody, the samples were stained with DAPI to reveal the nuclei. Cells incubated without primary antibody were regarded as negative controls. The cells were examined using confocal microscopy (FV1000, Olympus).

### Statistical analysis

The data were expressed as the means ± SD unless indicated otherwise. Comparisons between groups were performed using the two-tailed Student's *t* test. Pearson's χ^2^ test or Fisher's exact test was used to analyze the relationship between MIF expression and clinicopathological features. An ROC curve was generated for the sensitivity and specificity as a predictor of distant metastasis after obtaining the accumulated points of each sample. The differences between groups were compared using analysis of variance. SPSS 16.0 software (SPSS Inc.) was used for all statistical analyses. *P* values less than 0.05 were considered significant.

## SUPPLEMENTARY FIGURES AND TABLE


