# Supplementary material for: MIF, secreted by human hepatic sinusoidal endothelial cells, promotes chemotaxis and outgrowth of colorectal cancer in liver prometastasis
Source: Oncotarget. 2015 Jun 2;6(26):22410–23. doi: 10.18632/oncotarget.4198 (PMC4673172; doi:10.18632/oncotarget.4198)
Supplement: Supplementary file 1 [file oncotarget-06-22410-s001.pdf]

SUPPLEMENTARY FIGURES AND TABLE

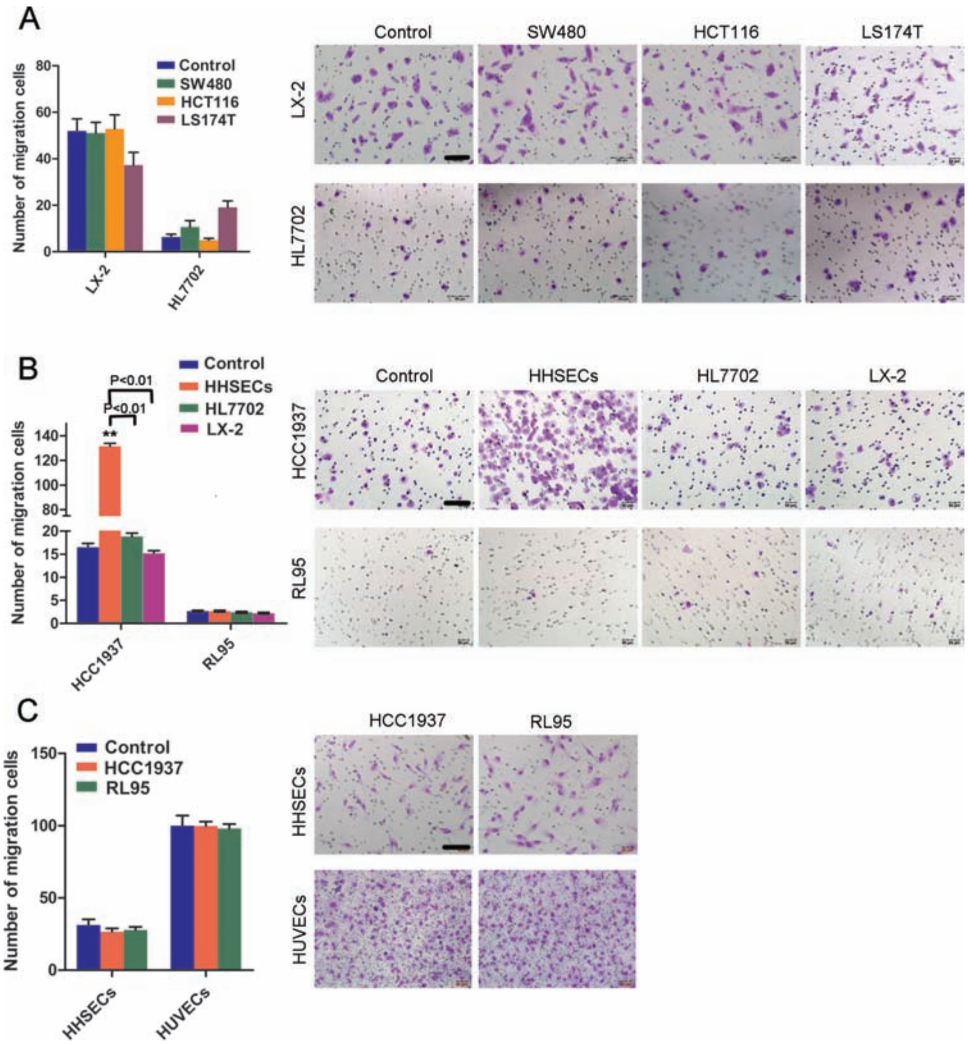

Supplementary Figure S1: Migration of target or non-target organ cells chemotracted by CRC cells. A. Transwell migration activity of the LX-2 and HL7702 cells chemotracted by CRC cells (compared to control) and representative images of migrated LX-2 or HL7702 cells chemotracted by CRC cells. Scale bar, 100 µm. **B.** Transwell migration activity of HCC1937 and RL95 cells chemotracted by HHSECs or HL7702 or LX-2 cells (compared to control) and representative images of migrated HCC1937 and RL95 cells chemotracted by HHSECs or HL7702 or LX-2 cells. Scale bar, 100 µm. **C.** Transwell migration activity of HHSECs and HUVECs chemotracted by HCC1937 or RL95 cells (compared to control) and representative images of migrated HHSECs and HUVECs chemotracted by HCC1937 or RL95 cells. Scale bar, 100 µm. Data are the means ± *SD* from three independent experiments. ***P* < 0.001 compared with control. *P* < 0.01 between groups.

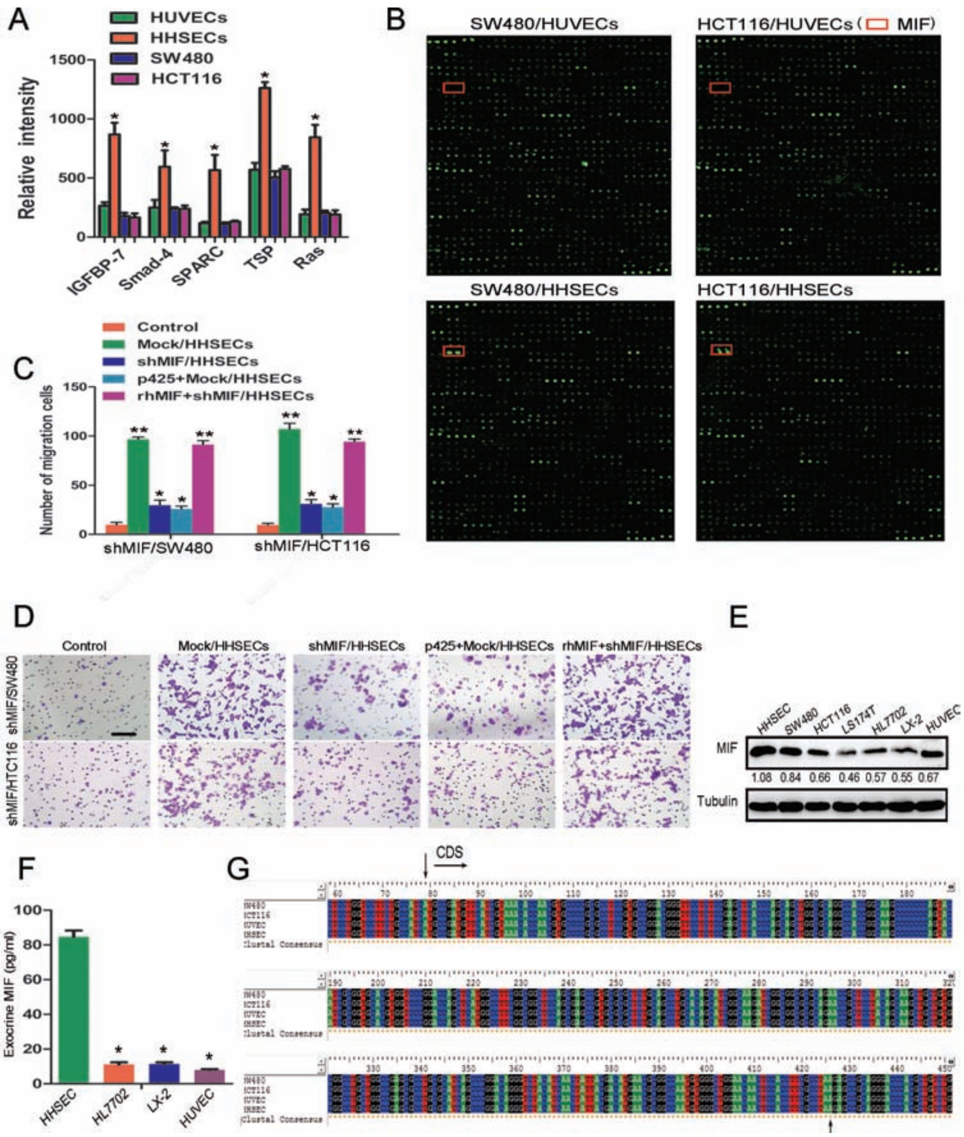

Supplementary Figure S2: MIF mediates the chemotraction of shMIF/CRC cells by HHSECs. A. Protein array assay (Raybiotech) was used to probe the expression of IGFBP-7, Smad 4, SPARC, TSP, and Ras in different conditioned media. $*P < 0.01$ compared with conditioned media of HUVECs. **B.** Protein array assay detected the MIF in SW480/HUVECs, SW480/HHSECs, HCT116/HUVECs and HCT116/ HHSECs conditioned media. **C.** Transwell migration activity of shMIF/SW480 and shMIF/HCT116 cells induced by different conditioned media. $*P < 0.01$, $**P < 0.001$ compared with control. **D.** Representative images of shMIF/SW480 and shMIF/HCT116 migration. Scale bars, 100 µm. Data are the means ± *SD* from three independent experiments. **E.** Western blot analysis of intracellular MIF. **F.** MIF secreted by cells was detected by ELISA. $*P < 0.001$ compared with HHSECs. **G.** The matched alignment of predicted DNA sequences of MIF after RT-PCR amplification in SW480 and HCT116 cells and HUVECs and HHSECs.

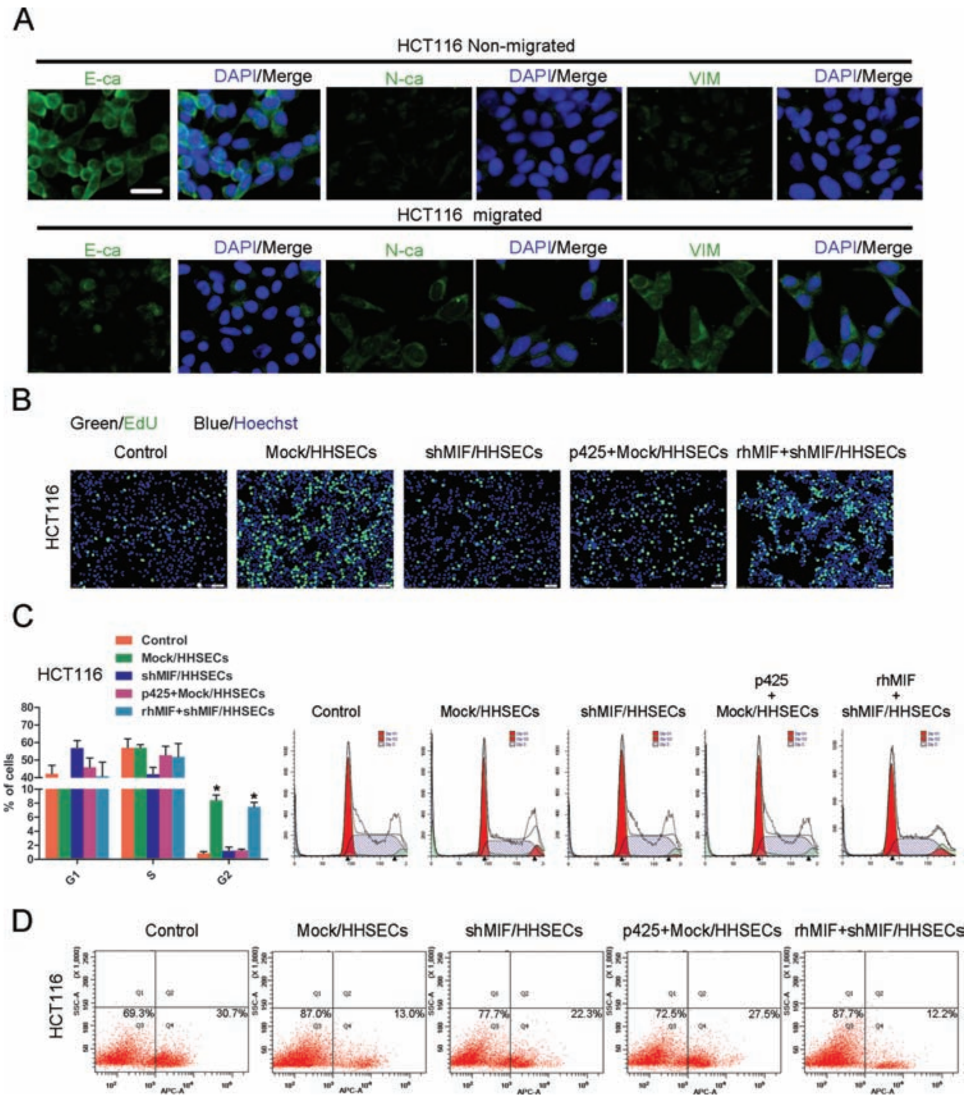

Supplementary Figure S3: Secreted MIF promotes EMT, proliferation, and apoptotic resistance of HCT116 cells. **A.** Immunofluorescence: migrated and non-migrated HCT116 cells following chemotaxis with HHSECs expressed E- and N-cadherin and vimentin. Scale bar, 20 μm. **B.** EdU cell proliferation assay of HCT116 cells. Scale bar, 100 μm. **C.** Cell cycle analysis of HCT116 cells grown in different conditioned media. MIF induced G2 phase arrest in HCT116 cells. **D.** 5-FU-induction of apoptosis was inhibited in HCT116 cells cultured with conditioned media containing MIF. Data represent means ± *SD* from three independent experiments. *$P < 0.01$ compared with control.

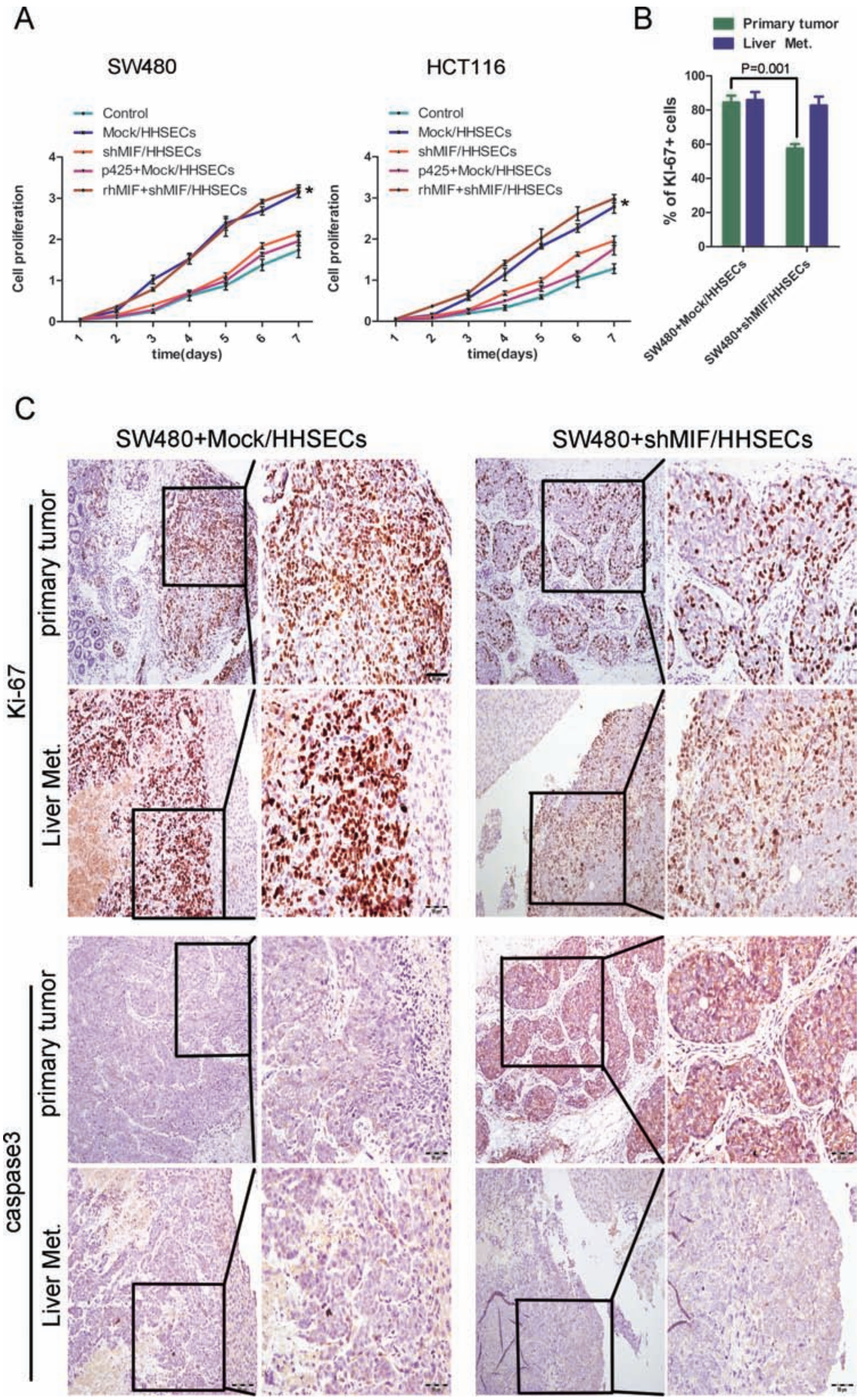

Supplementary Figure S4: MIF released from HHSECs promotes CRC cell proliferation. A. The proliferation of CRC cells cultured with conditioned media was assayed by CCK8. $^*P < 0.05$ compared with control. **B.** The Ki-67 cell proliferation index in different xenograft tumors and liver metastasized tumors. **C.** Immunohistochemical staining of Ki-67 and caspase3. Scale bars represent 50 μm.

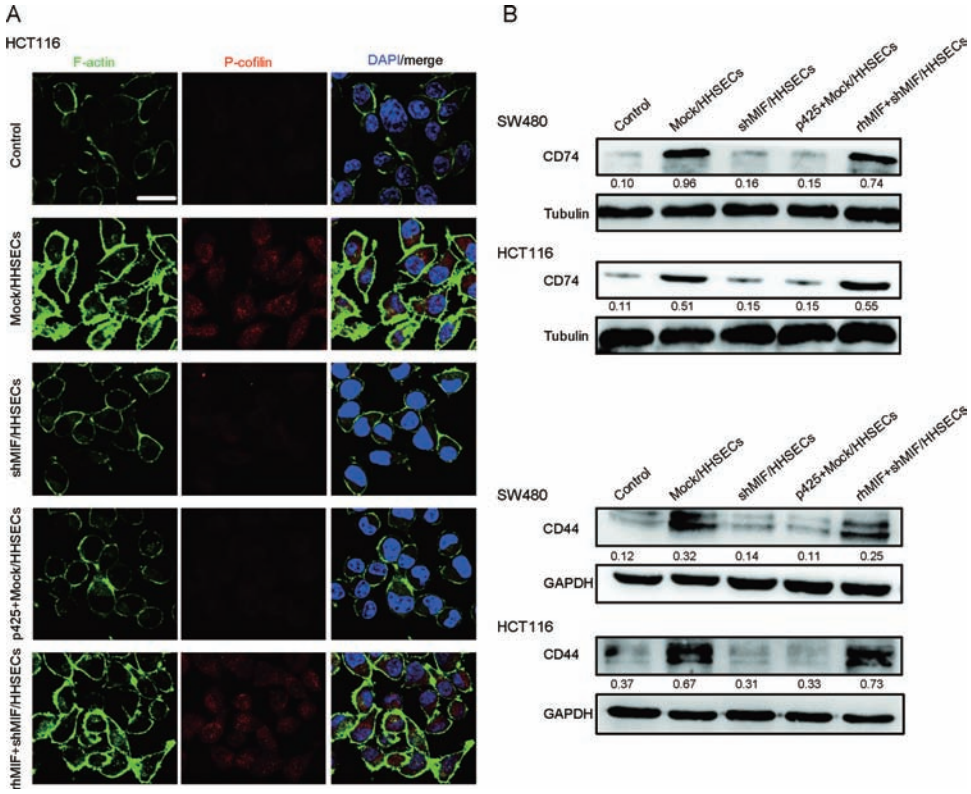

Supplementary Figure S5: MIF released from HHSECs regulates cytoskeletal protein, CD74 and CD44 expression. **A.** Immunofluorescence images of p-cofilin and F-actin expression in CRC cells cultured with different conditioned media. Scale bar, 20 µm. **B.** Western blot analysis of the CD74 and CD44 receptors of MIF in CRC cells.

Supplementary Table S1. Relationship between MIF immunoreactivity and clinicopathological factors in patient with CRC (*n* = 229)

Characteristics	No. patients, %	MIF immunoreactivity		*P* value
		Low, *n* = 162 (%)	High, *n* = 67 (%)	
Age				0.862
≤60y	121(53)	85(52)	36(54)	
>60y	108(47)	77(48)	31(46)	
Gender				0.412
Male	146(64)	106(65)	40(60)	
Female	83(36)	56(35)	27(40)	
Tumor grade				0.473
T1	82(35.8)	53(33)	29(43)	
T2	127(55.5)	95(59)	32(48)	
T3	17(7.4)	12(7)	5(7)	
T4	3(1.3)	2(1)	1(1)	
LN metastasis				0.093
Negative	167(73)	113(70)	54(81)	
Positive	62(27)	49(30)	13(19)	
Distant metastasis				0.968
Negative	126(55)	89(55)	37(55)	
Positive	103(45)	73(45)	30(45)	
Differentitation				0.160
Well	69(30)	51(31)	18(27)	
Moderate	123(54)	81(50)	42(63)	
Poor	37(16)	30(19)	7(10)	
Hist. classification				0.768
Adenocarcinoma	210(92)	148(91)	62(93)	
Muc. carcinoma	19(8)	14(9)	5(7)	
Survival time				0.061
<60M	104(45)	80(49)	24(36)	
≥60M	125(55)	82(51)	43(64)	
Status				0.263
Censored	141(62)	96(59)	45(67)	
Death	88(38)	66(41)	22(33)	

Note: LN = lymph node; Histo. classification = Histological classification; Muc. carcinoma = Mucinous carcinoma.
